# The effects of economic deprivation on psychological well-being among the working population of Switzerland

**DOI:** 10.1186/1471-2458-6-223

**Published:** 2006-09-04

**Authors:** Stefan Vetter, Jerome Endrass, Ivo Schweizer, Hsun-Mei Teng, Wulf Rossler, William T Gallo

**Affiliations:** 1 Center for Disaster and Military Psychiatry, University of Zurich, Zurich, Switzerland; 2 Department of Justice, Psychiatric-Psychological Service, Zurich, Switzerland; 3 Department of Epidemiology and Public Health, Yale University School of Medicine, New Haven, USA; 4 Psychiatric University Hospital of Zurich, Research Unit for Clinical and Social Psychiatry, Zurich, Switzerland

## Abstract

**Background:**

The association between poverty and mental health has been widely investigated. There is, however, limited evidence of mental health implications of working poverty, despite its representing a rapidly expanding segment of impoverished populations in many developed nations. In this study, we examined whether working poverty in Switzerland, a country with substantial recent growth among the working poor, was correlated with two dependent variables of interest: psychological health and unmet mental health need.

**Methods:**

This cross-sectional study used data drawn from the first 3 waves (1999–2001) of the Swiss Household Panel, a nationally representative sample of the permanent resident population of Switzerland. The study sample comprised 5453 subjects aged 20–59 years. We used Generalized Estimating Equation models to investigate the association between working poverty and psychological well-being; we applied logistic regression models to analyze the link between working poverty and unmet mental health need. Working poverty was represented by dummy variables indicating financial deficiency, restricted standard of living, or both conditions.

**Results:**

After controlling other factors, restricted standard of living was significantly (p < .001) negatively correlated with psychological well-being; it was also associated with approximately 50% increased risk of unmet mental health need (OR = 1.55; 95% CI 1.17 – 2.06).

**Conclusion:**

The findings of this study contribute to our understanding of the potential psychological impact of material deprivation on working Swiss citizens. Such knowledge may aid in the design of community intervention programs to help reduce the individual and societal burdens of poverty in Switzerland.

## Background

Recent statistics have indicated that working individuals represent a growing proportion of Swiss residents living in poverty [1,2], with an increase in the percentage of the working poor from 5% in 1995 to 7.5% in 1999. In a country with seemingly no modern historical record of indigence, and per-capita Gross Domestic Product (GDP) nearly 18% higher than the average of all OECD nations [3], the notion of working poverty is somewhat of a phenomenon. The mounting number of active labor force members who are economically insecure is, therefore, cause for concern among Swiss government leaders and policy makers, who acknowledge the potential obligations of social and economic services to counteract the consequences of chronic economic deprivation.

The association between poverty and health has been the focus of vast research [4], and includes both ecological studies linking income inequality to population health [5], and person-level investigations describing a relationship between socioeconomic deprivation and adverse outcomes, including mental health disorders [6-12]. A thorough, sex-stratified descriptive analysis of the correlates of poverty in Switzerland, based on the Swiss Household Panel (SHP) data, was published by Budowski and colleagues [13].

The potential health impact of *working *poverty has been the subject of less directed research, although numerous inquiries have examined occupational and social correlates of income inequality among employed individuals, building on the concept of relative deprivation first identified in the economics literature [14,15] and explored in more recent research [16,17]. Such studies have reported that lower economic status is associated with unstable work environments, high perceived job insecurity and threat of termination, and low levels of satisfaction with family, social life, and leisure [18-21]. There is also somewhat limited evidence [22,23] of a link between working poverty and health. Nevertheless, these cross-sectional studies are not designed to establish causation, largely because the temporal precedence of the exposure is not controlled, but also because of strong feedback relationships. It is therefore possible that many of the previously studied "outcome" variables motivate the observed association. For example, rather than poverty's provoking poor health, it is equally plausible that poor health leads to less intensive labor force participation, which results in financial deficiency. This financial deficiency could, in turn, affect health.

A number of other gaps remain in the literature. First, the majority of Swiss studies of the working poor are descriptive in nature and based on civil administrative data, which typically include only demographic information. They are thus largely ineffectual for research involving outcomes related to well-being. Second, no study attempting to associate working poverty with psychological health of which we are aware has been carried out in Switzerland, notwithstanding the emergence of this important class of Switzerland's poor. And finally, many studies have conventionally defined poverty based solely on income thresholds, which exclude the notion of deprivation, and thus do not fully capture the multifaceted nature of economic insecurity.

Regarding this last point, it is worth noting that the way in which we characterize poverty in this study is obviously dependent on the country under study–or perhaps more importantly, its level of economic development. Poverty is relevant only in context, with substantial definitional variation across nations according to economic circumstances [24]. Thus, although the lack of a home computer might be construed as deprivation in a relatively wealthy nation such as Switzerland, this would not be so in a developing country, where such an item would be considered a non-essential luxury good.

The objective of this research is to investigate the association between working poverty and two mental health measures among prime-age (20–59 years) workers in Switzerland. Using individual-level data from the 1999, 2000, and 2001 Swiss Household Panel, and a measure of poverty that takes account of both income deficiency and restricted standard of living [13,25], we assess whether working poverty status is associated with psychological well-being and unmet mental health need relative to the working non-poor.

We put forward two main hypotheses for this research:

*Hypothesis 1: *First, we propose that working poverty will be correlated with poorer psychological health and higher likelihood of unmet need, after accounting for socio-demographic and occupational characteristics, which may confound the relationship between poverty and mental health.

*Hypothesis 1a: *Working poverty, when defined by both income deficiency and restricted standard of living, will have a more salient effect on our outcomes than will working poverty, as defined independently by income deficiency or restricted standard of living.

*Hypothesis 2: *Second, we hypothesize that the effect of working poverty on psychological well-being will vary across demographic and occupational characteristics, where differential effects will be detectable by civil status, employment status, and sex. In particular, we purport that working poverty will have a more significant impact on psychological well-being among: unmarried individuals than those who are married or partnered, because of the lack of buffering provided by the social and financial support of a spouse; full-time employees than part-time workers, due to differences in labor force attachment; and women than men, because of the various interrelated burdens attached to the labor market and family (e.g. child care, sex discrimination, etc.).

## Methods

### Study design and data source

This is a cross-sectional study that uses data from the first 3 waves (1999–2001) of the Living in Switzerland survey of the Swiss Household Panel, a longitudinal survey designed to investigate trends in social dynamics among the Swiss population. The SHP is a nationally representative random sample of the permanent resident population, drawn on the basis of the largest national telecommunication provider's electronic telephone directory, which covers over 95% of all private households. Data are collected annually by telephone, and are obtained at both the individual and household level. All interviews are carried out in one of three (German, French, and Italian) of Switzerland's four national languages. At the 1999 SHP baseline, the sample comprised 7799 participants, who were aged 14 years and older, from 5074 households. The Swiss Household Panel is a collective effort of the Swiss National Science Foundation, the Swiss Federal Statistical Office, and the University of Neuchatel. A detailed description of the SHP is provided elsewhere [26]. As the SHP data are publicly available, and confidentiality is protected by identity masking, this study was exempt from institutional review board ethical assessment.

### Study sample

Our eligible study sample comprised prime-age individuals who reported working at either the 1999, 2000, or 2001 surveys (n_99 _= 4560; n_00 _= 4236; n_01 _= 3909). Eliminating observations with missing data in one or more study variables resulted in a final study sample of 5453 unique respondents, who contributed of 11869 person-wave data records. As nearly 20% of household income data were missing, we used a multiple imputation process [27] to obtain 4 imputed values, and then created 4 data sets, each of which contained one of the imputed values. Other missing data (6.7% of total sample) were largely the result of non-response to deprivation items and occupation-specific questions. Wave-level observations were combined (concatenated) in a repeated measures design to maximize statistical power in the analysis of psychological well-being. The Wave 1 (1999) subsample was used to analyze unmet mental health need, as one of the components required to construct this outcome variable was asked only at the 1999 survey wave.

### Dependent variables

Psychological well-being: The primary dependent variable in this study was a global measure of psychological well-being, taken from the World Health Organization Quality of Life Survey (WHOQOL-100) [28]. This variable was constructed based on responses to the SHP survey question, *Do you often have negative feelings such as having the blues, being desperate, suffering from anxiety or depression, if 0 means "never" and 10 "always"? *The 11-point response replaced a five-point response in the original, WHOQOL-100 question. Higher values represent worsening of psychological well-being.

Unmet mental health need: This binary (0, 1) dependent variable was coded as 1 if individuals jointly offered *psychological well-being *scores = 3 (top 25% of the distribution), and reported that they had not received mental health counselling in the year prior to the survey. Determination of mental health counselling was based on responses to the following survey question, *During the last 12 months, have you been treated for psychological problems?*

### Working poverty

The measure of working poverty, adapted from the designation of poverty developed by Budowski and colleagues for the SHP data [13], is defined by two dimensions: financial deficiency and restricted standard of living. Financial deficiency is characterized by a relative poverty measure, namely household income less than 60% of the weighted (equivalized) OECD median household income. (The SHP household income variable is scaled by SHP data personnel, so that it is directly comparable to the OECD income data.) Restricted standard of living is described by material deprivation, or the lack of 2 or more of 10 items or activities that are considered necessary by the majority of the Swiss population. Items and activities include: go to dentist if needed, color television, car (private use), holidays away from home (week/year), saving 100 Swiss Francs monthly, home with garden or terrace, dishwasher, washing machine (private use), savings in 3^rd ^(private) pillar of pension system, and home computer. Two additional items (monthly invitation of friends, monthly meal at restaurant) were excluded from consideration because of their potential endogenous determination by the outcomes. That is, psychological well-being might predict whether a respondent participates in such activities.

**Figure 1:**
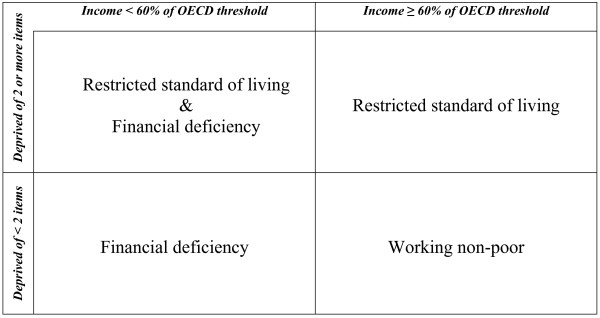
Categorizing working poverty: Classification of the working poor and non-poor.

Four, mutually exclusive dichotomous dummy variables represent our working poverty categories (Figure 1). The first variable (financial deficiency) characterized individuals residing in households with income below the OECD threshold, but who do not report lacking 2 or more items; the second variable (restricted standard of living) described individuals who lacked 2 or more items or activities, but who reported income above the OECD threshold; the third variable (both financial deficiency and having restricted standard of living) included individuals who reported both restricted standard of living and financial deficiency; and the fourth variable indicated respondents who reported neither restricted standard of living nor financial deficiency. This final variable, omitted in the analysis as the referent category, describes the working non-poor.

### Covariates

Adjustment variables and potential confounders were selected from a number of domains for use in multivariable cross-sectional models of the relationship between working poverty and our outcomes. These variables (Table 1) included demographic controls (sex, age, civil status, education, primary language spoken), health status (self-rated health), and occupational factors (self-employed, full-time employed, occupation, job prestige, risk of unemployment).

### Statistical analyses

To test our first hypotheses, we fit a multivariable model of working poverty to our outcomes (psychological well-being and unmet mental health need) with the 3 mutually exclusive dummy variables defining our poverty categories (working non-poor omitted as referent category), and compared the relative magnitude and statistical significance of the estimated coefficients on the working poor dummies. To test our second hypothesis, i.e. that the association between working poverty and our outcomes may vary within sub-populations, we added to the initial model a number of additional terms which multiplicatively interact the working poverty variables with relevant covariates, and assessed the statistical significance of the interaction terms. While effect modification was hypothesized for civil status, sex, and employment status, and these variables were thus the primary focus of such analyses, other covariates were also assessed for differential impact. As none of those variables was judged to be a significant effect modifier, the results of these supplementary analyses are not reported.

We used repeated measures Generalized Estimating Equations (GEE) [29-31] to investigate the association between working poverty and psychological well-being. GEE is a statistical method designed to correct for intra-subject correlation arising from repeated measures taken from the same individuals, as is the case in our study, wherein participants contribute up to 3 data records corresponding to the 1999–2001 surveys. Time, designated by survey year, was controlled in the GEE models; the interaction of time with all working poor categories was also investigated to rule out variation in the effect of working poverty on psychological well-being over time (i.e. across survey waves).

The analysis of unmet mental health was carried out with logistic regression, in which both bivariate and multivariable models were estimated. Once again, as a major component of this variable (i.e. treatment for psychological problems) was assessed just once, at the baseline survey, only the 1999 sub-sample of participants is used in this analysis.

For both outcomes, models were independently fit on 4 data sets, each of which contained one of the imputed household income values. We then averaged estimated coefficients and standard errors on explanatory variables for the 4 models, using the approach developed by Schafer and Olsen [27], to arrive at the reported results.

## Results

### Sample description

Sample members average 39 years of age, with 60% percent reporting married civil status (Table 2). Consistent with the full SHP cohort, the analytic sample is almost evenly divided by sex. The primary language spoken by sample members is German (67%), followed by French (28%), and Italian (5%). Regarding occupation, 61% of participants work full-time, and over 11% report self-employment. Occupational representation is roughly half (51%) management or professional, about one-fifth (22%) manual, unskilled, and agricultural, and just over one-quarter (combined) clerical and service. Four-hundred twenty-one individuals (7.7%) met our criterion for financial deficiency, without restricted standard of living; 404 (7.4%) were defined as having a restricted standard of living, without financial deficiency; 213 (3.9%) met both criteria, and 4415 (81%) met neither (working non-poor). Nineteen percent of sample members were defined as having unmet mental health need. The average psychological well-being score for the full sample was 1.66 (std. dev. = 1.98).

Comparing sample characteristics across poverty categories, we find a number of striking differences. Swiss workers with restricted standard of living are more often divorced, female, and French speaking than the non-poor. The occupational profile of the deprived also differs from that of the non-poor, with a higher proportion of workers in manual and lower-skilled occupations, and a lower proportion of workers in managerial and professional positions. Workers classified by low income also contrast markedly with the non-poor in several attributes. Income deficient sample members are more apt to be married (and less likely to be single), be self-employed, and once again, to work in lower-skill occupations than the working non-poor.

### Multivariable results

#### Psychological well-being

The results of multivariable estimation of the effect of working poverty on psychological well-being are presented in Table 3. Results are presented for a fully adjusted model without interaction terms (Hypothesis 1), the identical model with the additional interaction terms (Hypothesis 2), and stratified results by sex, the variable for which we find differential effects in the restricted standard of living measure of working poverty.

Our findings suggest that, after other factors are controlled, restricted standard of living is significantly (*p *< .001) negatively associated with psychological well-being relative to the working non-poor, confirming Hypothesis 1, whereas financial deficiency is not. The combined effect of the two variables, just marginally non-significant (*p *< .10), is smaller in magnitude than the independent effect of deprivation, reflecting the statistical averaging of the two effects, contrary to Hypothesis 1a. Among covariates, female sex and higher perceived risk of unemployment are also negatively related to psychological well-being. Significant protective factors include being married, better educated, German or French speaking, and full-time employed, and rating health as very well or well.

Considering the model testing effect modification, our results indicate sex differences (*p *< .05) in the association between restricted standard of living and psychological ill-health, with women demonstrating a more potent effect than men. Neither of the other hypothesized effect modifiers (employment status, marital status) was significant. Hypothesis 2 is therefore partially confirmed. The results of the sex-stratified analyses illustrate what cannot be demonstrated by the interaction model: namely, that the psychological well-being of both sexes is significantly influenced by the experience of working poverty.

#### Unmet mental health need

In Table 4, we present the results of logistic analysis of unmet mental health need. Both unadjusted odds ratios, and those adjusted for covariates, are presented. As no significant effect modifiers were identified in the unmet need estimations, refuting Hypothesis 2, we do not provide results for the models that included interaction terms. Our results once again suggest a significant effect of restricted standard of living, supporting Hypothesis 1. In particular, we find that the unadjusted risk of unmet need (i.e. jointly reporting poor psychological health and no psychological treatment in the past 12 months) for participants with restricted standard of living is more than double (Odds ratio [OR] = 2.28; 95% Confidence Interval [CI] 1.75 – 2.95) that of the working non-poor. This result is somewhat attenuated by the addition of covariates. The fully-adjusted model indicates approximately 55% added risk of unmet mental health need associated with restricted standard of living (OR = 1.55; 95% CI 1.17 – 2.06), after adjusting for other factors. The variable representing the combined states of financial deficiency and restricted standard of living is not significant in both the unadjusted and adjusted models, once more contradicting Hypothesis 1a. As in the analysis of psychological health, we find no evidence of an association between financial deficiency (independently) and unmet mental health need.

## Discussion

In this cross-sectional study, we investigated whether poverty is concurrently associated with psychological well-being among working residents of Switzerland, and whether the assumed relationship varied by relevant subgroups. We then assessed whether poverty affects the obtaining of mental health care for those individuals with the most critical need for such treatment. Our findings suggest that of the two independent definitions of poverty applied to our data, restricted standard of living, a measure intended to proxy material deprivation, has a discernable negative relationship with both psychological well-being, in general, and the likelihood of having had mental health counseling among study participants whose (negative) well-being scores were in the top quartile of the distribution, which we assumed to reflect a necessity for psychological services. The results further indicate sex differences in the effect of economic deprivation on overall psychological well-being. Thus, while the data confirm that the psychological health of both Swiss men and women is adversely influenced by living without certain common household items, or partaking in fairly customary activities, women appear to be affected more intensely than men.

The analysis did not suggest that relative income deficiency had any measurable bearing on psychological health or unmet counseling need. Furthermore, the intersection of restricted standard of living and income deficiency, assumed to be the worst possible state of working poverty, was only not significantly related to psychological ill-health, owing to the dilution of effect caused by combining a strongly significant factor (deprivation) with a non-significant one (low income). In any case, the reader should be reminded that, given the cross-sectional design of the study, all reported associations merely imply correlation, and should not be interpreted as causal.

Our findings from this research are generally supportive of those of earlier studies linking material deprivation to negative psychological outcomes, and strongly consistent with evidence from the Poverty and Social Exclusion (PSE) Survey of Britain [32], with one noteworthy exception. That is, whereas the items identified by the deprived in the PSE research-which are perhaps the underlying motivation for the observed mental health effect-differed by sex, in our study they did not. British women were at elevated risk for poor psychological health when they were unable to afford the cost of practical goods, or those with collective benefit to the family, such as two pairs of all weather shoes, redecorating the home, or repairing/replacing items such as furnishings and electrical goods; on the contrary, men with low mental health function were distinguished by being unable to afford to spend money (weekly) on themselves.

In contrast, our data revealed that among Swiss workers with a restricted standard of living (deprived), men and women reliably designated the same 2 items from the potential list of 10: the inability to save 100 Swiss Francs monthly, and to contribute to the 3rd pillar superannuation fund. Consequently, the sex difference in the magnitude of effect of material deprivation in Switzerland seems rather produced by sex-specific perception and processing of the deprivation than by variation in the goods or activities. The environment in which the deprivation occurs may also play a role in the observed sex differences. Single parenthood, more likely among women than men in the sample of working poor, is associated with multiple burdens (e.g. job, children, household duties) and less time for recreational activities, which could limit the opportunity for emotionally supportive social relationships. Recent research [33] has suggested that such relationships are substantially more protective against depression for women than for men.

Sex differences aside, the notion that Swiss workers with a restricted standard of living are so defined by items of a somewhat non-material nature is intriguing from a sociological perspective, and may imply a uniquely Swiss economic outlook. Both of the items commonly identified by deprived individuals (i.e. those with the poorest psychological well-being) acknowledge the importance of current financial behavior to future consumption. Further considering that low income, when combined with deprivation, had no additional effect on psychological health in our analyses, one could infer that the association between working poverty and poor mental health is based more on the inability to accumulate wealth, at least part of which may not be expended until retirement, than on any deficiency in current consumption goods or services, or the ability to purchase them. Such farsighted underpinnings differ markedly from the current items identified by British PSE study subjects with elevated mental health scores. It would be imprudent, however, to wholly attribute the cross-national difference to cultural divergence in perception, as deprived PSE participants included both working and unemployed individuals.

It would be similarly unwise to ignore the fact that the mechanism between poverty and health is a complex one, and that large secondary data sets such as the SHP, no matter their depth or sophistication, can never fully capture the process of mediation from a socioeconomic status to an adverse health status or event, if that is, in fact, the direction of the relationship. Our findings should be interpreted in the context of this limitation. Seemingly simpler matters, moreover, remain unresolved. For example, much debate has focused on the question of how best to operationalize poverty for the purpose of investigating its correlates. Studies have used a range of measures, including relative income levels, earnings thresholds for receipt of public assistance, and varied measures of socio-economic stratification and labor market exclusion. However, no real consensus exists on the optimal measure of poverty. In this study, which exclusively considered employed individuals, we both separated and combined variables measuring low income and deprivation to broaden the possibilities of potential associations. Having found an association in just one of the two variables, and a weakening of this effect when uniting the two measures, appears to justify our decision.

Two other matters also merit mention. First, our primary outcome variable has not been validated against clinical assessment of mental ill-health, as the establishment of psychological illness prevalence and incidence is not a goal of the SHP. Further, the outcome variable captures neither intensity nor severity. Rather, this variable is a subjective pseudo-frequency of negative psychological states that summarizes individuals' perception of their psychological well-being. The robust statistical results nonetheless suggest that our measure is sensitive to the experience of deprivation. And second, as our design is cross-sectional, we are unable to assess whether the association between restricted standard of living and poorer psychological well-being is a causal one, and must be left for future research.

## Conclusion

This research may have important implications. From our findings, social planners and policy makers can be made aware that financial deprivation among working Swiss may have mental health consequences. Mental ill-health, with its myriad associated costs, has the potential to place a great burden on the economic and social structure of Switzerland. Being able to identify a population that is at risk for mental illness can provide a base for further research. Future investigations should focus more closely on the specific needs, attitudes, and expectations of the economically deprived residents of Switzerland. A more specific knowledge regarding the mental health consequences of financial deprivation can help in designing intervention programs in the community, and thus help to reduce the individual and societal burden of poverty in Switzerland.

## Competing interests

The author(s) declare that they have no competing interests.

## Authors' contributions

SV participated in the design of the study, helped draft the manuscript, and oversaw all work on the project. JE performed the data management, participated in the statistical analysis, and helped draft the manuscript. IS participated in the design of the study and helped draft the manuscript. HT led the statistical analysis, interpreted the findings, and helped draft the manuscript. WR participated in the design of the study and provided critical revisions to the manuscript. WTG conceived the study, participated in its design, oversaw the statistical analysis, and drafted the manuscript. All authors read and approved the final manuscript.

## Pre-publication history

The pre-publication history for this paper can be accessed here:



## Figures and Tables

**Table 1:** Variable Descriptions

Variable Name	Variable Description
Female	Binary variable for sex: 1 = female, 0 = male
Age	Continuous variable representing biological age in years
Married, Divorced, Single	Binary dummy variables for civil status: Married: 1 = married, 0 otherwise Divorced: 1 = divorced, separated, widowed, 0 otherwise Single: 1 = single, never married, 0 otherwise (referent variable)
Education	Binary variable: 1 = apprenticeship & more; 0 = no formal education
German, French, Italian	Binary dummy variables for primary language spoken: German: 1 = German, 0 otherwise French: 1 = French, 0 otherwise Italian: 1 = Italian, 0 otherwise (referent variable)
Full-time	Binary variable for type of employment: 1 = full-time (100%); 0 = part-time
Self-employed	Binary variable for type of employment for current job: 1 = self-employed, 0 = works for employer
Risk of unemployment	Continuous variable: risk of unemployment scale in next 12 months 0 (no risk at all) – 10 (a real risk)
Treimans prestige scale	Continuous variable: Treimans prestige scale for main job, 0 (lowest prestige) – 100 (highest prestige)
Professional, Clerical, Service, Other	Binary design variables for occupation, main current job: Professional: 1 = legislators, senior officials, managers, professionals, 0 otherwise Clerical: 1 = clerks, 0 otherwise Service:1 = service workers, market sales workers, 0 otherwise Other: 1 = skilled agricultural & fishery workers, plant and machine operator assemblers, elementary occupations, armed forces, 0 otherwise (referent category)
Health status	Binary variable for self-assessed health status: 1 = feeling very well/well right now, 0 = average, not very well/not well at all

**Table 2:** Descriptive statistics for full sample and by working poverty classification

Variable	Full Sample (N = 5453)	Restricted Std. of Living (N = 404)	Financially Deficient (N = 421)	Non-poor (N = 4415)
*Dependent Variables*				
Psychological well-being	1.66 (1.98)	2.62 (2.45)	1.49 (1.97)	1.56 (1.89)
Unmet mental health need ^†^	19%	33%	19%	18%
*Socio-demographic Factors*				
Age (in years)	38.95 (10.56)	37.04 (10.55)	39.78 (10.00)	39.10 (10.61)
Female Sex	51%	58%	53%	50%
Education (apprentice or more)	81%	77%	76%	82%
Married	60%	46%	73%	60%
Divorced	10%	20%	10%	9%
Single	30%	34%	17%	31%
German	67%	49%	69%	70%
French	28%	44%	25%	26%
Italian	5%	7%	6%	4%
*Occupational Factors*				
Full-time	61%	58%	49%	64%
Self-employed	11%	10%	15%	10%
Professional	51%	37%	34%	55%
Clerical	14%	16%	11%	14%
Service	13%	19%	15%	12%
Other	22%	28%	40%	19%
Risk of unemployment (0–10)	1.79 (2.54)	2.37 (2.95)	1.84 (2.63)	1.70 (2.46)
Treimans Prestige Scale (0–100)	44.53 (13.27)	40.57 (12.47)	40.04 (12.47)	45.66 (13.23)
*Self-perceived health status*				
Health (healthy/very healthy)	87%	79%	86%	89%

Non-percentage table values represent mean (standard deviation). ^†^Based on Wave 1 (1999) sample of 4184 participants.

**Table 3:** Association of working poverty with psychological well-being: Full sample and sex-stratified results

Variable	Full Sample (N = 5453)	Full Sample With Interactions	Women (N = 2773)	Men (N = 2680)
*Working Poverty*				
Financially deficient (FD)	-.01 (.08)	-.01 (.08)	-.03 (.15)	.01 (.10)
Restricted standard of living (RSL)	.63 (.09)***	.19 (.27)	.71 (.14)***	.46 (.13)***
Both FD & RSL	.21 (.13)	.21 (.14)	.18 (.20)	.18 (.21)
Working non-poor	*Ref*.	*Ref*.	*Ref*.	*Ref*.
*Interactions*				
RSL × Married	--	-.21 (.21)	--	--
RSL × Divorced	--	.04 (.27)	--	--
RSL × Full-time	--	.41 (.22)	--	--
RSL × Female	--	.52 (.23)*	--	--
*Adjustment Variables*				
Age	-.003 (.002)	-.003 (.003)	-.004 (.004)	-.002 (.003)
Female Sex	.40 (.05)***	.35 (.06)***	--	--
Education (apprentice or more)	-.10 (.04)*	-.10 (.04)*	-.10 (.06)	-.11 (.06)
Married	-.31 (.06)***	-.29 (.06)***	-.42 (.09)***	-.16 (.07)*
Divorced	.14 (.09)	.13 (.09)	.12 (.13)	.15 (.14)
Single	*Ref*.	*Ref*.	*Ref*.	*Ref*.
German	-.68 (.11)***	-.68 (.12)***	-.72 (.18)***	-.63 (.16)***
French	-.24 (.12)*	-.24 (.13)	-.18 (.19)	-.31 (.17)
Italian	*Ref*.	*Ref*.	*Ref*.	*Ref*.
Full-time	-.26 (.05)***	-.29 (.05)***	-.24 (.08)**	-.48 (.09)***
Self-employed	-.004 (.07)	-.004 (.07)	.19 (.11)	-.15 (.08)
Professional	.08 (.09)	.08 (.09)	-.12 (.17)	.17 (.11)
Clerical	.01 (.09)	.01 (.09)	-.20 (.16)	.27 (.13)*
Service	-.004 (.08)	-.01 (.09)	-.13 (.14)	.04 (.13)
Other	*Ref*.	*Ref*.	*Ref*.	*Ref*.
Risk of unemployment	.10 (.01)***	.10 (.01)***	.09 (.01)***	.10 (.01)***
Treimans Prestige Scale	-.002 (.002)	-.002 (.002)	-.001 (.004)	-.002 (.003)
Health (healthy/very healthy)	-1.24 (.07)***	-1.23 (.08)***	-1.45 (.10)***	-.93 (.11)***
Intercept	3.15 (.23)***	3.20 (.24)***	3.97 (.35)***	2.82 (.33)***

Table values represent estimated coefficient (standard error). *p < .05; **p < .01; ***p < .001. All model specifications were adjusted for time.

**Table 4:** Association of working poverty with unmet mental health need using Wave 1 (1999) subsample (*N = 4184*)

Variable	Unadjusted Model Odds Ratio (95% CI)	Model Adjusted for Covariates Odds Ratio (95% CI)
*Working Poverty*		
Financially deficient (FD)	1.10 (.82 – 1.48)	0.87 (.55 – 1.41)
Restricted standard of living (RSL)	2.28 (1.75 – 2.95)***	1.55 (1.17 – 2.06)**
Both FD & RSL	1.42 (0.93 – 2.17)	1.11 (.73 – 1.69)
Working non-poor	--	*Ref*.
*Adjustment Variables*		
Age	--	.99 (.98 – .99)*
Female Sex	--	1.71 (1.40 – 2.08)***
Education (apprentice or more)	--	.90 (.72 – 1.40)
Married	--	.82 (.67 – 1.01)
Divorced	--	.92 (.68 – 1.25)
Single	--	*Ref*.
German	--	.47 (.33 – .65)***
French	--	.70 (.50 – .99)*
Italian	--	*Ref*.
Full-time	--	1.03 (.85 – 1.25)
Self-employed	--	1.22 (.93 – 1.58)
Professional	--	1.15 (.80 – 1.64)
Clerical	--	1.20 (.84 – 1.70)
Service	--	1.33 (.97 – 1.83)
Other	--	*Ref*.
Risk of unemployment	--	1.07 (1.04 – 1.10)***
Treimans Prestige Scale	--	1.00 (.99 – 1.01)
Health (healthy/very healthy)	--	.37 (.31 – .46)***

*p < .05; **p < .01; ***p < .001
